# Diagnosis and surgical treatment of obstructed hemivagina and ipsilateral renal anomaly in a dog: a case report

**DOI:** 10.3389/fvets.2024.1488107

**Published:** 2024-12-23

**Authors:** Younwoo Choo, Jun-Sik Cho, Ah-Young Cha, Hwi-Yool Kim, Jung-Moon Kim

**Affiliations:** ^1^Department of Veterinary Surgery, College of Veterinary Medicine, Konkuk University, Seoul, Republic of Korea; ^2^Department of Veterinary Surgery, Sky Animal Medical Center, Yongin, Republic of Korea

**Keywords:** dog, Gartner duct cyst, Herlyn–Werner–Wunderlich syndrome, OHVIRA, unilateral renal agenesis

## Abstract

Obstructed hemivagina and ipsilateral renal agenesis (OHVIRA), also called Herlyn–Werner–Wunderlich syndrome, is an extremely rare Müllerian duct anomaly accompanied by Wolffian duct anomalies. A 10-year-old intact female Yorkshire Terrier weighing 3.35 kg was presented with anorexia, depression, vomiting, and abdominal pain. Radiography, ultrasonography, and computed tomography revealed uterine didelphys, obstructed hemivagina, a cystic structure around the right uterus, and right renal agenesis, leading to the diagnosis of OHVIRA syndrome. An ovariohysterectomy and decompression of the obstructed right hemivagina were performed, and the cystic structure near the right uterine horn was removed by *en bloc* resection, along with the right uterus and ovary. After the surgical intervention, the patient’s symptoms including abdominal pain, anorexia, and depression were immediately resolved. The patient was followed up for 1 month postoperatively with ultrasonography at 2-week intervals, which revealed the progression of mild fluid retention in the right hemivagina. However, no additional urogenital findings were identified, and the patient continued to exhibit no overt clinical symptoms. This case report describes the diagnosis and surgical treatment of the first documented case of OHVIRA syndrome in an animal. Unlike in human medicine, where vaginal septectomy is performed to prevent dilatation of the obstructed hemivagina and thereby resolve clinical symptoms, performing ovariohysterectomy combined with fluid aspiration from the obstructed hemivagina showed a favorable postoperative prognosis in the dog.

## Introduction

Obstructed hemivagina and ipsilateral renal agenesis (OHVIRA), also called Herlyn–Werner–Wunderlich syndrome, is a rare developmental abnormality with anomalies in both the paramesonephric (Müllerian) ducts and the mesonephric (Wolffian) ducts (1). OHVIRA syndrome is characterized by a triad of uterus didelphys, obstructed hemivagina, and ipsilateral renal agenesis (2). OHVIRA syndrome typically presents with lower abdominal pain during the menstrual cycle (1). Dysmenorrhea and dyschezia have also been reported (3). Hematometrocolpos or hematocolpos resulting from an obstructed hemivagina can cause acute onset of abdominal pain, fever, and vomiting (4). If an infection occurs, it may progress to pyocolpos or pyometrocolpos, leading to symptoms such as fever, chills, or vomiting (2). Even in the cases where hemivaginas are incompletely obstructed, OHVIRA syndrome may be delayed or manifest as an asymptomatic condition (3).

Treatment of OHVIRA syndrome varies, but in human medicine, vaginal septectomy (86.5%) is the most preferred surgical method performed (5). The purpose of surgical techniques is to alleviate the obstruction of the hemivagina and improve the clinical symptoms while causing minimal damage to the function and structure of the urinary and reproductive systems (6).

To the author’s knowledge, there have been no reports on OHVIRA syndrome diagnosed in veterinary medicine. This case report describes the clinical signs, diagnosis based on imaging findings, and surgical intervention of a canine patient with OHVIRA.

## Case description

**Figure 1:**
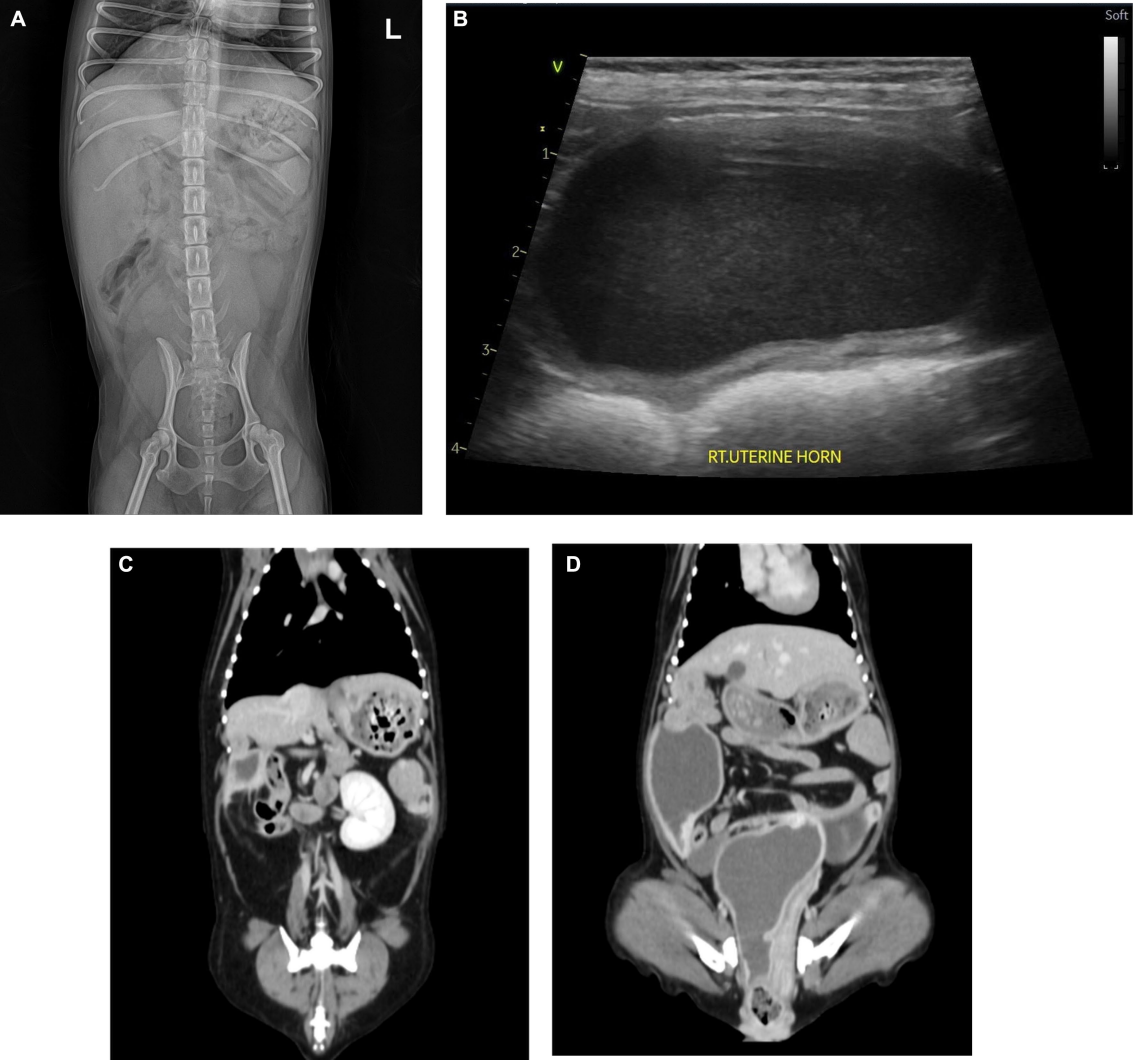
Radiograph **(A)**, ultrasonography **(B)**, and coronal CT image **(C,D)** of the abdomen. Urogenital malformations. **(A)** A dilated tubular structure was found along the right abdominal wall in the lower abdomen. **(B)** Markedly expanded right uterine horn filled with hyperechoic fluid. **(C)** Right-sided renal agenesis. The left kidney is found without compensatory enlargement. **(D)** CT scan revealed dilated right uterus and obstructed right hemivagina. In addition, a cystic lesion was found around the right uterine horn.

A 10-year-old intact female Yorkshire Terrier weighing 3.35 kg was presented with clinical signs of anorexia, depression, dysuria, dyschezia, and vomiting. Upon physical examination, the dog showed a severe abdominal pain on palpation. Complete blood count (CBC), C-reactive protein (CRP), canine pancreatic lipase immunoreactivity (cPLI), and blood gas analysis were within normal ranges. The radiographic examination revealed a dilated tubular structure in the lower abdomen, along the right abdominal wall. The ultrasonography found a distinctly dilated right uterine horn filled with hyperechoic fluid. A cystic structure measuring approximately 1.8 × 2.1 cm was observed between the uterine horns without continuity to surrounding organs, such as uterine horn or urinary bladder. Right kidney was not detected either in radiography or ultrasonography. The dog underwent CT imaging under general anesthesia to confirm US findings and determine the extent of adhesions. The dog was induced with anesthesia with butorphanol and alfaxalone and maintained using isoflurane. The CT scan revealed a dilated right uterine horn, a cyst around the right uterine horn, uterine didelphys, expanded right hemivagina with obstruction, and right renal agenesis, suggesting OHVIRA syndrome (Figure 1). Based on the clinical symptoms and imaging findings, pyometra was suspected, and the dog was recommended an immediate surgical resection (ovariohysterectomy).

**Figure 2:**
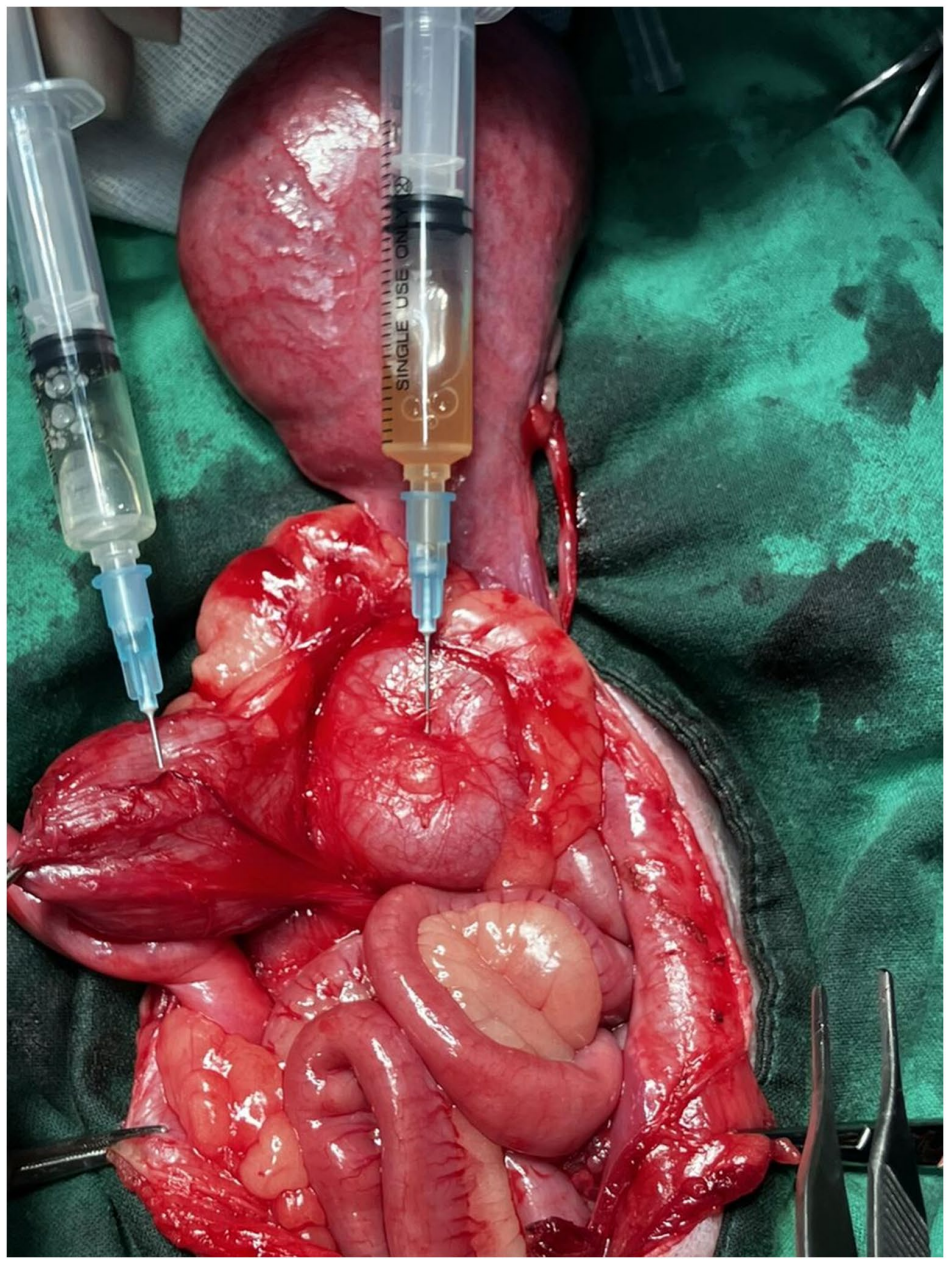
Fluid aspirated from the urinary bladder (left) and cystic structure (right). The fluid aspirated from the cystic structure between the two reddish, fluid-filled structures exhibited a clear but darker yellow color compared to the urine.

After the CT imaging, the patient was moved to the operation room, and the laparotomy was performed. Prior to the surgical procedure, famotidine was administered as a premedication, and cefazolin was used as perioperative antibiotics. During laparotomy, fluid-filled structures were found around the urinary bladder with severe adhesion thereto, making them challenging to distinguish. To distinguish these structures, fluids were aspirated from each of the four structures, including the urinary bladder. Two aspiration samples contained the same cloudy reddish fluid, indicating that they originated from reproductive organs, such as the right uterine horn and obstructed hemivagina. The fluid aspirated from the structure between the two reddish fluid-filled structures was clear but darker yellow than the urine from the urinary bladder (Figure 2). This indicated that the structure was not continuous from the right uterus. The right kidney and its ureter were not found, confirming the CT findings.

**Figure 3:**
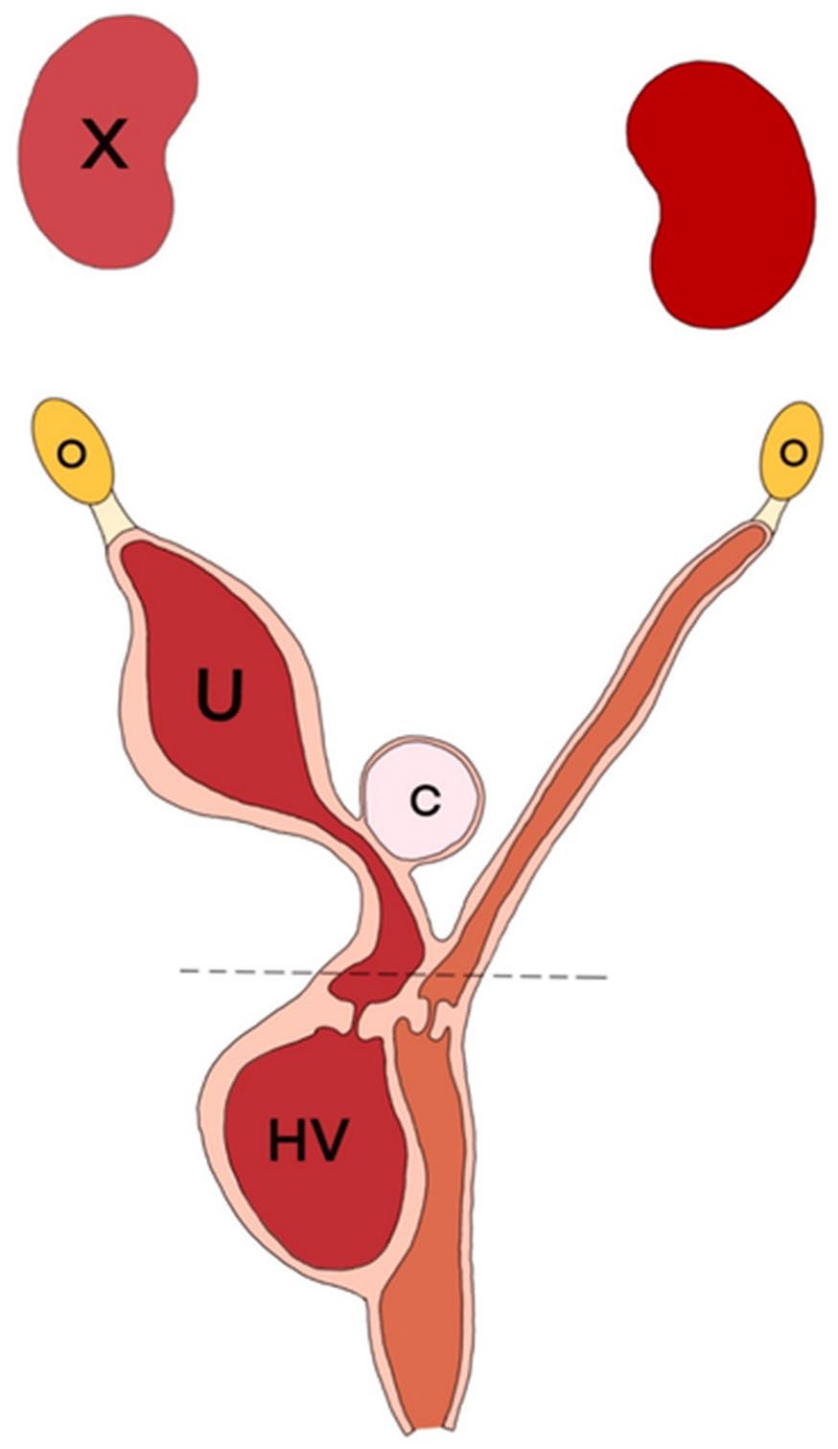
Anatomical schema of the findings from the operation in the ventrodorsal view. A cyst **(C)** located between the uterine horns (U) was resected with the right uterus and ovary (O). Decompression of obstructed hemivagina (HV) was performed via fluid aspiration. The dotted line is the resection line at the surgery.

Despite the severe adhesions among the abdominal organs, the ovariohysterectomy proceeded routinely. After resection of the suspensory ligament of the right ovary, the right uterus was resected using an electrothermal bipolar vessel sealing device at the level of the cervix along the uterine horn. The cyst was resected *en bloc* along with the right uterus and ovary. Similarly, ‘the left ovary and uterus were excised in a same manner (Figure 3). Decompression of the expanded obstructed hemivagina was performed. The abdominal wall was closed routinely. Clinical signs including dysuria, dyschezia, and anorexia were resolved immediately after surgery.

**Figure 4:**
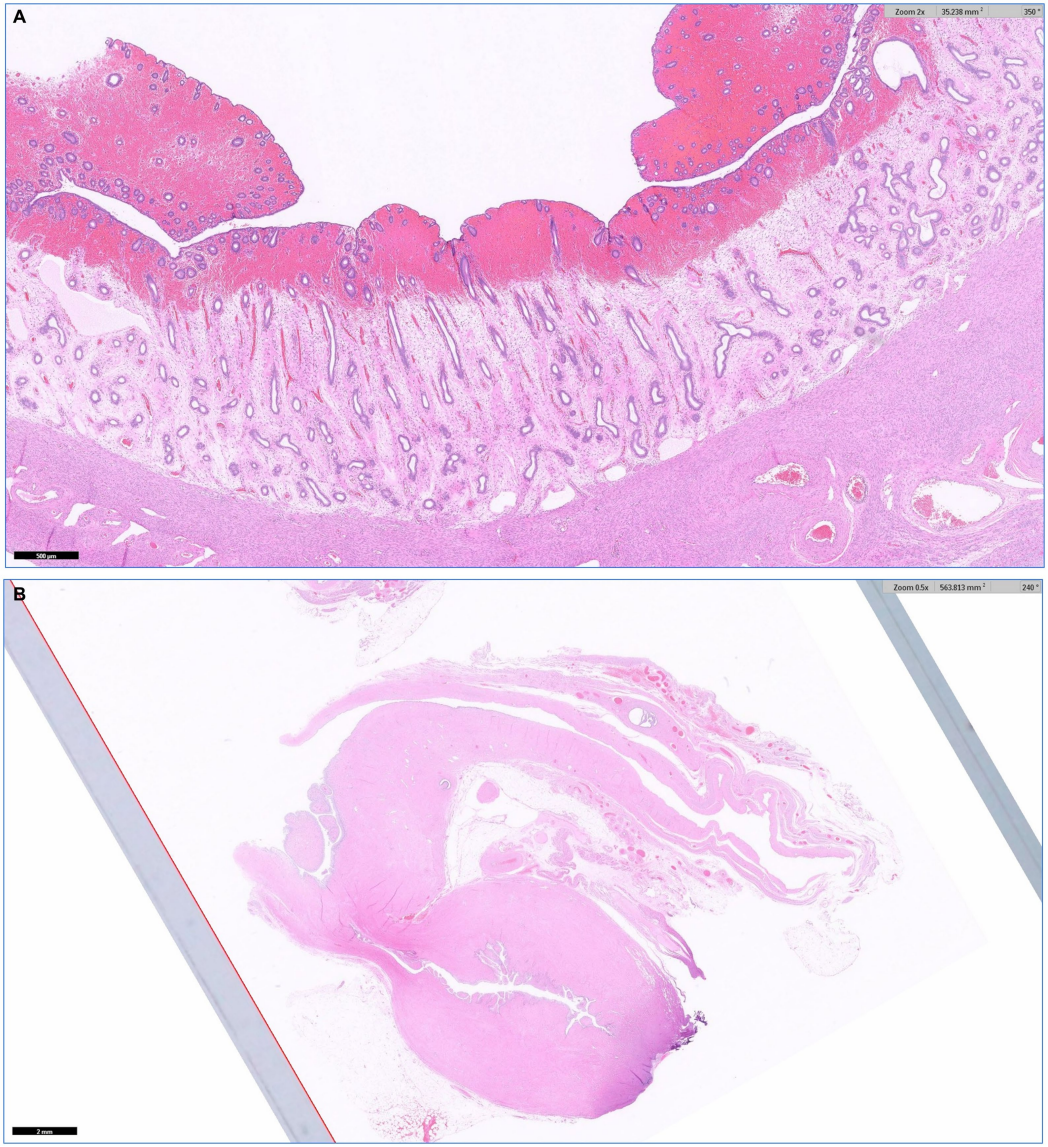
Histopathology of the right uterine by HE stain. **(A)** The right uterus exhibited mild endometrial hyperplasia with hemorrhage and mild glandular ectasia, without overt neoplasia or significant inflammation (no overt pyometra). **(B)** A mass with well-differentiated glandular structures and fibrous tissue around the cervix was identified, which is suggestive of an endocervical cyst or polyp.

A portion of the resected right uterine was referred for histologic analysis (IDEXX Laboratories). Histopathologically, the right uterus appeared with mild endometrial hyperplasia with hemorrhage and mild glandular ectasia. No overt neoplasia or a marked inflammatory component was found. In addition, a mass consisting of well-differentiated glandular structures and fibrous tissue was found around the area of the cervix, which was considered to be an endocervical cyst or endocervical polyp formation (Figure 4).

Postoperative ultrasonography showed dilated right hemivagina with the accumulation of fluid. The accumulated fluid appeared with debris. However, no fluid accumulation or dilation was noted in the left uterine remnant. A recheck was conducted at 2-week intervals using ultrasound examinations. Over the course of a month, mild progressive dilation of the right hemivagina was observed, with fluid and debris present within it.

## Discussion

OHVIRA syndrome is an extremely rare Müllerian duct anomaly accompanied by Wolffian duct anomalies, with incidence ranging from 0.1 to 3.8% of the female population in human medicine (1, 3, 5). The Wolffian duct, particularly its distal part, undergoes a critical development phase between 36 and 46 days of gestation in dogs (7). Since the ureteric bud originates from the Wolffian duct and the formation of the Müllerian duct is affected by this duct, malformations of the Wolffian duct may eventually lead to ipsilateral renal agenesis or malformations of the uterus and vagina (8). If the Wolffian duct does not degenerate properly, cystic remnants can form around the uterus and vagina, resulting in single or multiple Gartner duct cysts (9).

Imaging examinations play a crucial role in the precise diagnosis of OHVIRA. In human medicine, magnetic resonance imaging (MRI) is the gold standard for diagnosing Müllerian duct anomalies (MDA) due to its excellent soft tissue contrast (2). However when MRI is not available, CT scans can be carried out to confirm ultrasound findings and make a definitive diagnosis (1, 2). In the case of this dog, ultrasonography, CT scan, and intraoperative findings confirmed uterine didelphys, obstructed hemivagina, and right renal agenesis, which led to the diagnosis of OHVIRA syndrome. In addition, a solitary cystic structure around the right uterine horn was found in this dog. No communication was observed between the cystic structure and the right uterus. Although histologic examination of a cyst was not conducted, the cystic structure appeared to be a Gartner duct cyst, based on the location of the cyst, the serous fluid present within, and the patient’s history of MDA.

Some reports described clinical symptoms caused by obstructed hemivagina and Gartner duct cyst due to mechanical urethral or intestinal compression, such as dysuria, dyschezia, tenesmus, abdominal pain, and dyspareunia (10, 11). However, these symptoms may be delayed due to the regular menstrual flow from the unobstructed hemivagina or the presence of small communication in the septum between hemivaginas (1). This dog showed completely obstructed, blind hemivagina, thus falling into classification 1 (1.1, with blind hemivagina; 1.2, cervicovaginal atresia without communicating uteri) of OHVIRA’s two classifications (12, 13).

An obstructed hemivagina can lead to blood retention within the vagina, potentially causing hematocolpos or progressing to hematometrocolpos. This menstrual reflux may trigger chronic inflammation in the reproductive system and ultimately result in severe adhesions between abdominal organs (14). In the present case, laparotomy revealed severe pelvic adhesions. Fluid aspiration was performed to differentiate between the adhered organs. The aspiration of a reddish fluid indicated that the involved structures were part of the reproductive system, confirming blood retention due to the presence of a blind hemivagina. Histopathological analysis excluded pyometra, suggesting that the primary cause of these extensive adhesions may be the retention and retrograde flow of blood within the hemivagina and uterus.

In human medicine, the first choice for patients with OHVIRA is to excise the vaginal septum using an electrothermal bipolar vessel sealing device or holmium laser to achieve patency (86.5%) as this allows preservation of fertility and the hymen. Invasive procedures such as hemivaginectomy (2.2%) or hemihysterectomy (4.2%) are indicated only in highly complicated cases (5). In veterinary medicine, the female dogs and cats are recommended to be neutered to eliminate unwanted pregnancies and reduce the risk of mammary neoplasia (15). Therefore, in animal patients presenting with symptoms caused by OHVIRA, ovariohysterectomy can help alleviate symptoms due to OHVIRA and effectively prevent its recurrence.

This case report describes the diagnosis and surgical treatment of the first case of OHVIRA syndrome seen in a dog. In the present case, hematometrocolpos or hematocolpos resulting from an obstructed hemivagina caused abdominal pain, anorexia, and vomiting due to mechanical urethral or intestinal compression. An ovariohysterectomy with decompression of an obstructed right hemivagina was performed, and a cystic structure located around the right uterine horn was resected *en bloc* along with the right uterus and ovary. Through cystectomy, potential cause for urethral or intestinal compression was eliminated without risk of recurrence (16). Surgery resolved clinical signs immediately after surgery. However, as the hemivagina tissue persists and the septum remains intact, long-term follow-up is required to determine whether the obstructed hemivagina refills with fluid. Revision surgery such as vaginal septectomy may be required if symptoms recur due to the residual right hemivagina.

## Data Availability

The original contributions presented in the study are included in the article/Supplementary material, further inquiries can be directed to the corresponding author.

## Supplementary material

SUPPLEMENTARY FIGURE S1 Macroscopic findings. Severe adhesions were observed among the abdominal organs. Between the left uterine horn and the dilated right uterine horn, two cystic structures were identified. Before ovariohysterectomy, fluid aspiration was performed to differentiate between these structures.

SUPPLEMENTARY FIGURE S2 Incised right uterine horn after ovariohysterectomy. Reddish fluid within the lumen of the right uterine horn was identified.

SUPPLEMENTARY FIGURE S3 Fluid aspirated from right hemivagina. The cloudy reddish appearance of the fluid indicated that the structure originated from the reproductive organ.

SUPPLEMENTARY FIGURE S4 Coronal CT image of abdomen. Right hematometrocolpos was observed, resulting in the dilation of the right uterine horn and right hemivagina. This condition caused displacement of the urinary bladder toward the left side of the abdominal cavity.
