# Supplementary figures and images for: Diagnosis and surgical treatment of obstructed hemivagina and ipsilateral renal anomaly in a dog: a case report
Source: Front Vet Sci. 2024 Dec 23;11:1488107. doi: 10.3389/fvets.2024.1488107 (PMC11729922; doi:10.3389/fvets.2024.1488107)

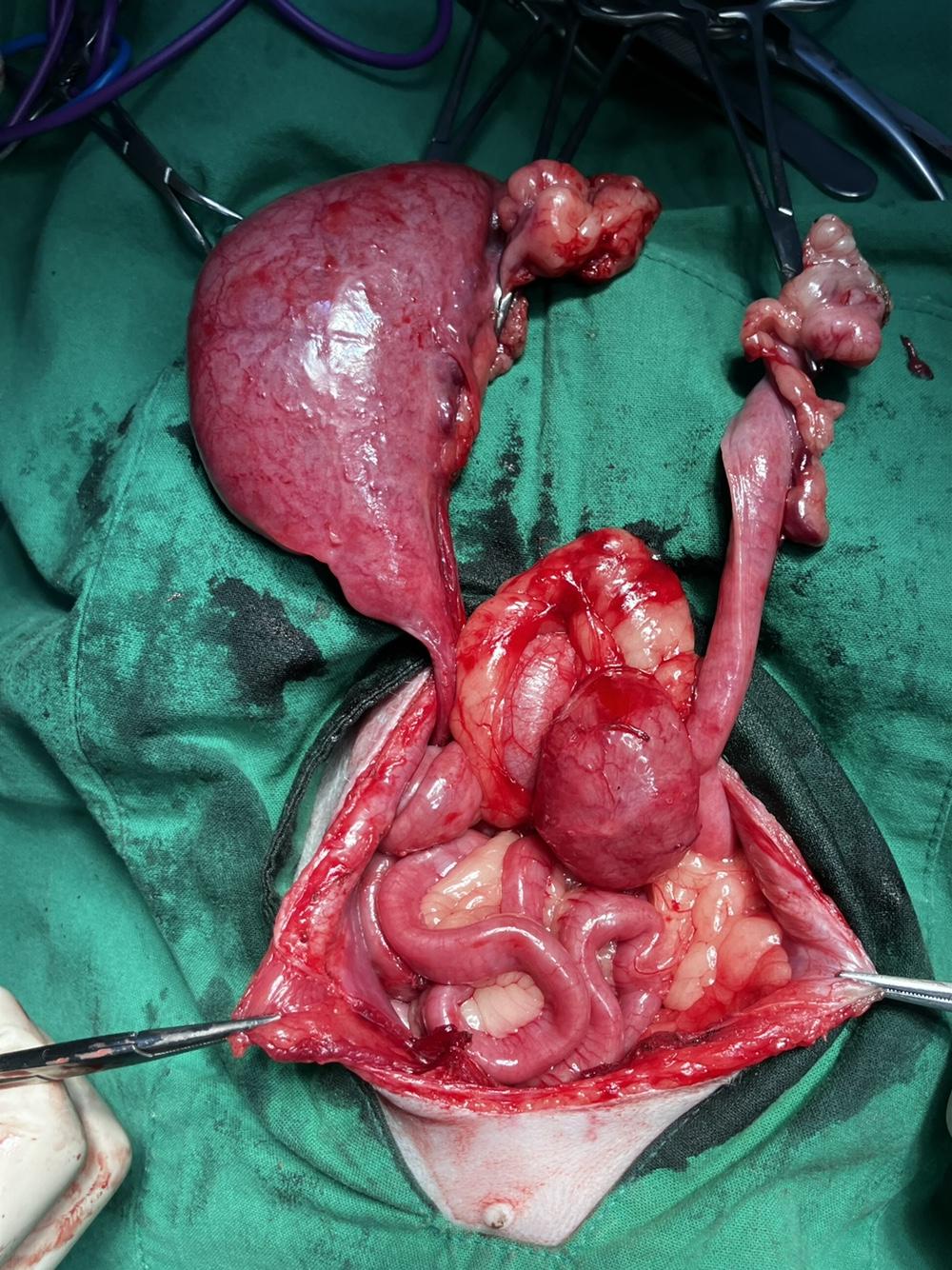

Supplement: SUPPLEMENTARY FIGURE S1 — Macroscopic findings. Severe adhesions were observed among the abdominal organs. Between the left uterine horn and the dilated right uterine horn, two cystic structures were identified. Before ovariohysterectomy, fluid aspiration was performed to differentiate between these structures. [file Image_1.JPEG]

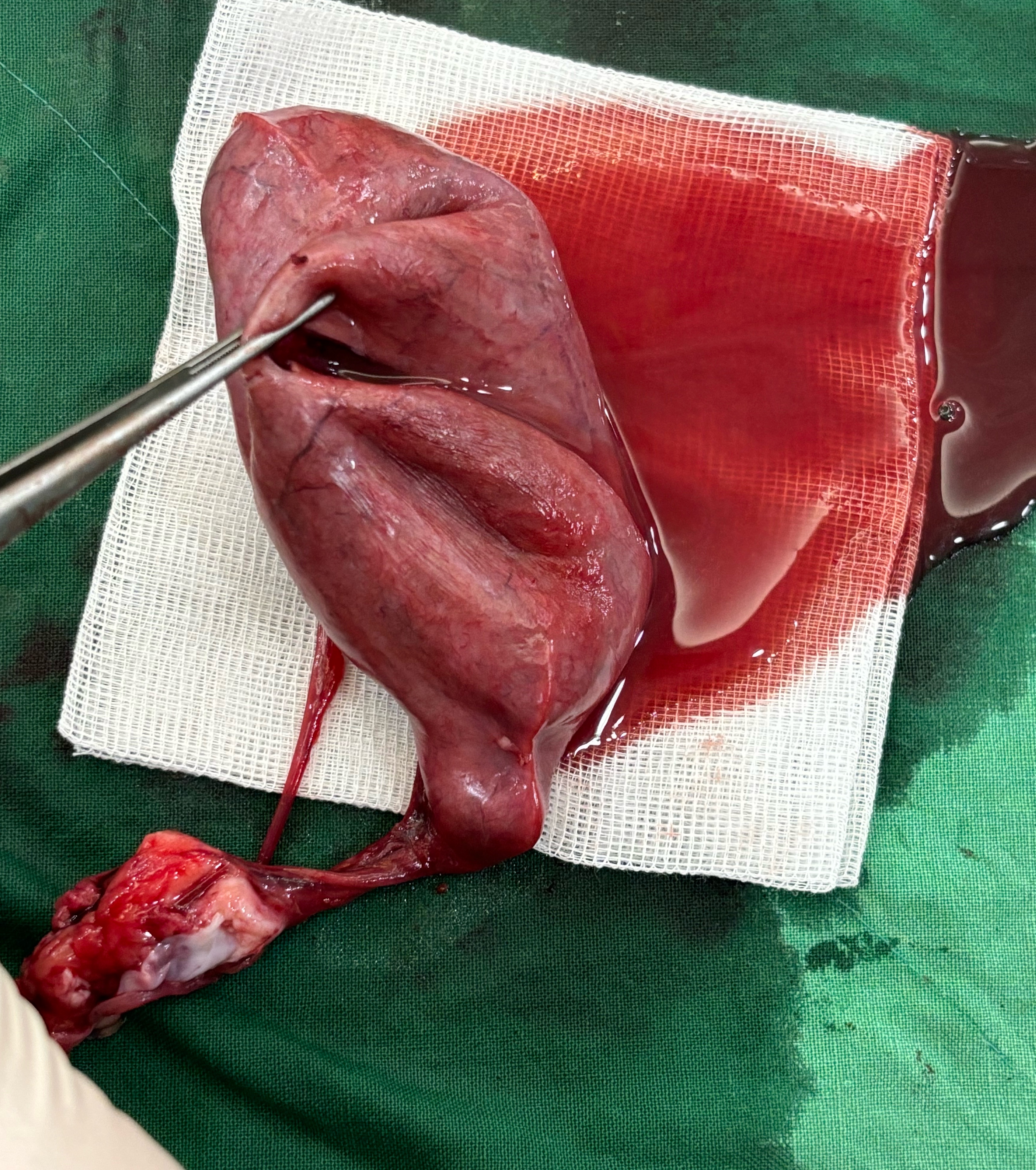

Supplement: SUPPLEMENTARY FIGURE S2 — Incised right uterine horn after ovariohysterectomy. Reddish fluid within the lumen of the right uterine horn was identified. [file Image_2.JPEG]

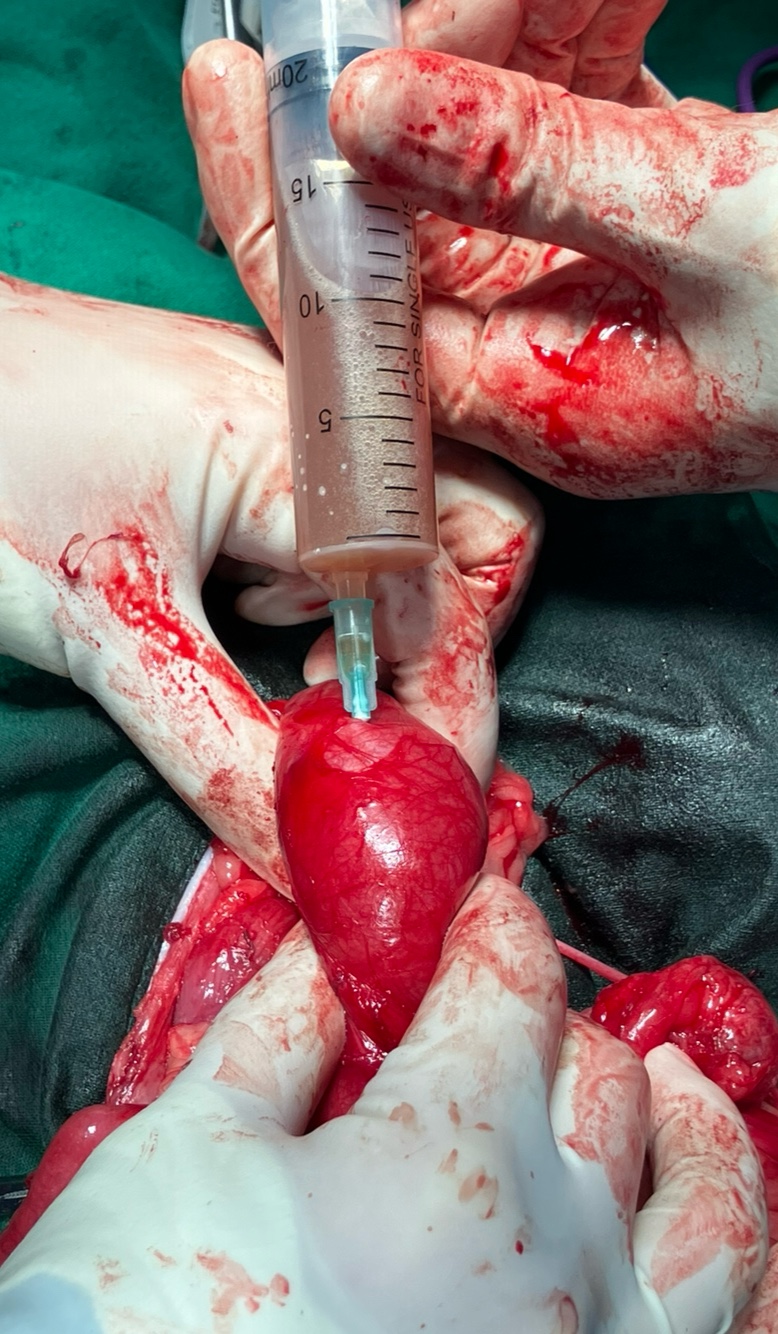

Supplement: SUPPLEMENTARY FIGURE S3 — Fluid aspirated from right hemivagina. The cloudy reddish appearance of the fluid indicated that the structure originated from the reproductive organ. [file Image_3.JPEG]

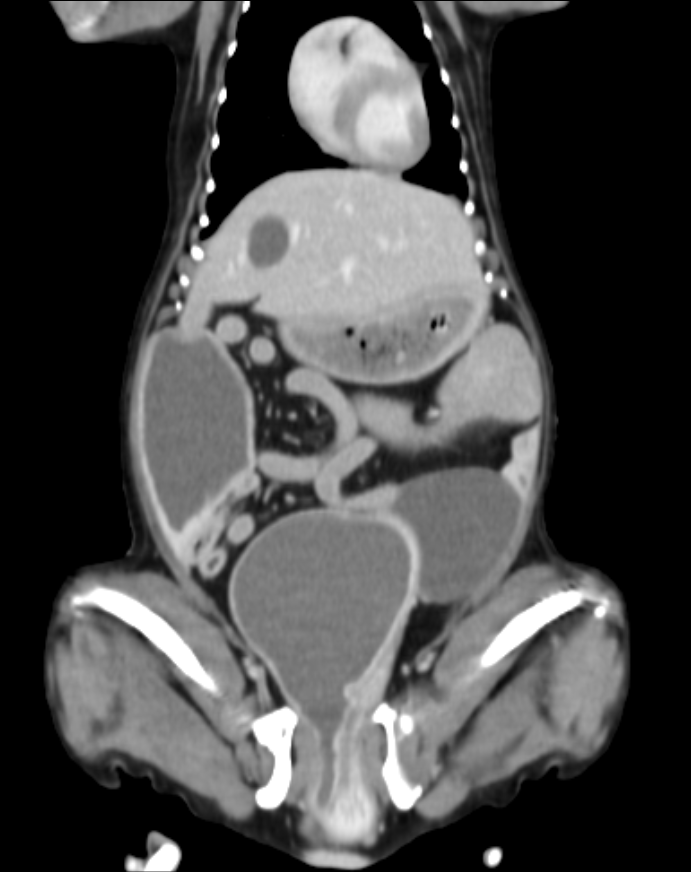

Supplement: SUPPLEMENTARY FIGURE S4 — Coronal CT image of abdomen. Right hematometrocolpos was observed, resulting in the dilation of the right uterine horn and right hemivagina. This condition caused displacement of the urinary bladder toward the left side of the abdominal cavity. [file Image_4.PNG]
